# Content of Patient Electronic Messages to Physicians in a Large Integrated System

**DOI:** 10.1001/jamanetworkopen.2024.4867

**Published:** 2024-04-04

**Authors:** Vincent X Liu, Pamela Kaercher, Jennifer Manickam, Eric Smallberg, Kanishka Bhutani, Michelle Mancha, Kristine Lee

**Affiliations:** 1 Division of Research, Kaiser Permanente, Oakland, California; 2 The Permanente Medical Group, Oakland, California

## Abstract

This quality improvement study describes the content of electronic health record messages from patients to physicians in a large integrated health care system using natural language processing algorithms.

## Introduction

Electronic health record adoption has produced tremendous growth in patient-physician inbox messages,^1^ which accelerated during the COVID-19 pandemic,^2,3^ with some health systems instituting charges for this mounting clinical burden.^4,5^ However, message content across populations is poorly understood, limiting systematic approaches that can improve message-related workloads.

## Methods

The Kaiser Permanente Northern California (KPNC) Research Determination Office deemed this quality improvement study to be exempt from review and did not require informed consent because it was not human participants research. We followed the STROBE reporting guideline.

In 2019, KPNC initiated the Desktop Medicine Program to operationalize a solution to address messaging volume and expeditiously direct patients to the optimal person, information, or service (eAppendix in Supplement 1). Program development integrated regional clinician teams and real-time natural language processing (NLP) algorithms to label message content and sort messages to appropriate roles (eg, teleservice representatives, medical assistants, pharmacists, physicians) before reaching individual physician inboxes. Category labels were added to the NLP output when specific Desktop Medicine response workflows were in place. The NLP pipeline was trained on a corpus of more than 20 000 messages annotated by nurses with triage experience (eAppendix in Supplement 1) and integrated within the electronic health record in May 2022 with bimonthly programmatic updates. The program initially focused on adult and family medicine messages and subsequently expanded to pediatric messages in October 2022. We examined the content and variability in message labels for adult and family medicine and pediatric physicians between April 1 and August 31, 2023, with descriptive statistics and coefficient of variation and quantified the association with physician inbox volume. Statistical analysis was performed using Python version 3.10.8 (Python Software Foundation), Pandas package version 1.5.3, and Microsoft Excel.

## Results

There were 4 709 261 patient messages during the study period, of which 3 655 065 (77.6%]) received at least 1 label, most commonly for medications (1 546 411 [32.8%]), skin conditions (438 631 [9.3%]), messages with attachments (389 372 [8.3%]), and emergent content for expedited review (359 144 [7.6%]) (Table). Overall, 1 374 309 messages (29.2%) included at least 2 labels, most commonly including skin conditions with medications (179 707 [4.1%]), skin conditions with attachments (171 955 [4.0%]), and emergent content with medications (148 357 [3.4%]).

**Table.** Proportion of Labels Assigned to Inbox Messages Based on the Desktop Medicine Natural Language Processing Pipeline^a^

Category	Count, No. (%) (N = 4 709 261)^b^
Medications	1 546 411 (32.8)
Skin conditions	438 631 (9.3)
Attachment	389 372 (8.3)
Emergent	359 144 (7.6)
Short done^c^	296 631 (6.3)
Letters	286 137 (6.1)
Laboratory order requests	285 350 (6.1)
Blood pressure	243 536 (5.2)
Respiratory symptoms	202 465 (4.3)
Controlled substances	148 569 (3.2)
Neck and back pain	145 577 (3.1)
Mental health	139 978 (3)
Immunizations and PPD	131 198 (2.8)
COVID-19 other	102 330 (2.2)
Urinary symptoms	96 718 (2.1)
Foot and ankle pain	91 334 (1.9)
Ear pain/ear wash	89 550 (1.9)
Sore throat	85 782 (1.8)
Knee pain	69 491 (1.5)
Diarrhea	54 090 (1.1)
Asthma	48 275 (1)
COVID-19 vaccine	46 219 (1)
COVID-19 therapeutics	36 142 (0.8)
Language^d^	29 120 (0.6)
Sexual health	24 217 (0.5)
FIT kit	21 870 (0.5)
COVID-19 test	13 315 (0.3)
Erectile dysfunction	13 268 (0.3)
Flu shot clinic	6650 (0.1)
Mpox	859 (0)
Questionnaire	37 (0)

Abbreviations: FIT, fecal immunochemical test; PPD, purified protein derivative.

^a^ The Desktop Medicine natural language processing pipeline including 31 content areas linked to operational and clinical workflows to optimize regional review and response of electronic messages.

^b^ Label proportions based on messages sent by patients to adult and/or family medicine physicians and pediatric physicians between April 1 and August 31, 2023. Messages can be labeled in more than 1 category.

^c^ Short done is a category used to describe messages which typically can be addressed quickly by teleservice representatives.

^d^ Language is a label applied to messages that include non-English content.

Weekly label variability (Figure) was lowest for controlled substances (coefficient of variation: 4.2 [95% CI, 3.2-6.1]), medication (4.5 [95% CI, 3.4-6.5]), neck and back pain (4.7 [95% CI, 3.6-6.8]), and erectile dysfunction (5.3 [95% CI, 4.1-7.6]) and highest for influenza vaccine (96.4 [95% CI, 73.8-139.3]), Mpox (43.1 [95% CI, 33.0-62.2]), and COVID-19 vaccine (35.2 [95% CI, 26.9-50.8]). Of 4 835 200 messages evaluated by regional Desktop Medicine staff, 1 541 657 (31.9%) were resolved by regional medical assistants or teleservice representatives, physicians, and pharmacists before reaching individual physician inboxes.

**Figure.**
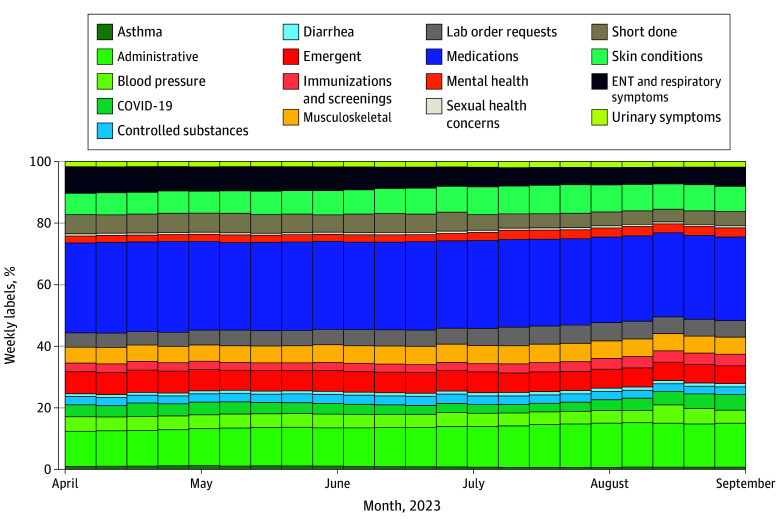
Weekly Proportion of Message Labels Within Category Groupings Used by the Desktop Medicine Program From April 1 to August 31, 2023 For visual simplicity, some labels have been grouped within larger categories. For example, COVID-19 includes labels related to COVID-19 therapeutics, vaccination, testing, and other. Musculoskeletal pain includes labels related to foot, ankle, back, neck, and knee pain. ENT indicates ear, nose, and throat.

## Discussion

This study including more than 4 million patient messages found novel insights that inform health system approaches for addressing the high volume of patient-physician electronic communications in a timely and effective fashion. First, identifying common and recurring categories (eg, medication-related or skin condition–related labels) across many physicians informs the development of population-level approaches for reducing individual physician inbox message volume. Second, quantifying the variability in message categories highlights acute changes in specific categories that can be used to identify emerging trends and facilitate rapid operational responses (eg, COVID-19, influenza, or Mpox). Third, real-time message analysis, labeling content before reaching a physician inbox for expedited regional review, can shorten the time to clinical assessment for potentially emergent conditions. Additionally, although most messages were felt to be most appropriately addressed by the intended physician recipient, the program was effective at resolving more than 1.5 million patient messages, accounting for nearly one-third of overall volume.

Study limitations included that the findings were from a single health care system and that the program did not include newer large language models (eg, GPT-4),^6^ which could improve content labeling and responses. Future enhancements are also needed to effectively address messages with multiple topics and those with no category labels. In conclusion, this study’s results suggest that a health system–wide approach to classifying patient messages paired with well-defined regional workflows can improve timely responses and substantially reduce physician inbox volume.

## Notes

Supplement 1. **eAppendix.** Supplemental Materials
eReferences

Supplement 2.
Data Sharing Statement
