# Supplementary material for: Content of Patient Electronic Messages to Physicians in a Large Integrated System
Source: JAMA Netw Open. 2024 Apr 4;7(4):e244867. doi: 10.1001/jamanetworkopen.2024.4867 (PMC11192179; doi:10.1001/jamanetworkopen.2024.4867)
Supplement: Supplement 1. — eAppendix. Supplemental Materials eReferences [file jamanetwopen-e244867-s001.pdf]

Supplemental Online Content

Liu VX, Kaercher P, Manickam J, et al. Content of patient electronic messages to physicians in a large integrated system. *JAMA Netw Open*. 2024;7(4):e244867.
doi:10.1001/jamanetworkopen.2024.4867

eAppendix. Supplemental Materials
eReferences

This supplemental material has been provided by the authors to give readers additional information about their work.

© 2024 Liu VX et al. *JAMA Network Open.*

eAppendix. Supplemental Materials

Description of the Desktop Medicine Program

In the early 2000s, Kaiser Permanente Northern California was at the forefront of integrating secure messaging into patient-physician communication. Initially, the system was expected to manage an average of five messages per day and was intentionally designed for simplicity, allowing patients to directly contact their primary care providers with free text in both the subject line and the body of the message. However, the landscape of healthcare evolved rapidly due to technologic advancements, paradigm shifts in healthcare delivery, and changing patient preferences, leading to a significant uptick in the adoption and use of this communication platform.

In the subsequent 15 years, the convenience of secure messaging communications has resulted in a substantial increase in message volume and become a major source of physician fatigue and challenge. The surge in messaging volume was also identified as a contributing factor to increased rates of physician burnout and attrition. In response to this challenge, KPNC initiated the 'Desktop Medicine' program, a strategic endeavor to optimize the routing and handling of secure messages across a highly integrated healthcare delivery system that, today, employs >9,000 physicians serving 4.5 million members across 21 medical centers and >200 clinics. The program aimed to alleviate the burden on primary care providers by directing specific patient inquiries to regional physicians and staff, seeking to jointly enhance physician well-being and improve patient outcomes, thereby, allowing primary care providers to concentrate their attention on more complex patient issues.

© 2024 Liu VX et al. *JAMA Network Open.*

However, one significant challenge in developing the Desktop Medicine program was the resource-intensive and variable process of categorizing and directing these messages written in natural language using a free-text format. To address this challenge, KPNC has deployed and iteratively refined the use of Natural Language Processing (NLP) and Machine Learning (ML) to improve the efficiency and accuracy of message processing (further described below). The implementation of these technologies led to several important enhancements that have improved the program's capabilities.

For example, close collaboration between nurses and regional Desktop Medicine pharmacists has produced more effective labeling of messages, particularly those containing specific medication-related topics. In addition, improvements in clinicians' assessment of the NLP labeling capabilities – both its strengths and limitations – have significantly improved overall training protocols. This training has highlighted opportunities to further refine the message labeling and validation process, maximizing the opportunities to efficiently identify the most appropriate follow-up actions for specific messages.

The integrated message analysis pipeline for Desktop Medicine

The NLP classification pipeline consists of a FastText[1,2] language model, a custom-built fuzzy string-matching model called catfuzz that is highly customizable and returns explainable results, a transformer-based DistilBERT[7] multilabel classification model that understands contextual meaning, and a simple neural network that weighs the predictions made by catfuzz and DistilBERT to return a final prediction. Before patient secure messages are processed by these models, the subject and body of the messages are concatenated then cleaned of rich text format strings, messy punctuation, and duplicated spacing. Responses to specific health questionnaires are then identified by specific strings at the beginning of the message and do not pass through the rest of the pipeline to avoid further mislabeling.

© 2024 Liu VX et al. *JAMA Network Open.*

Next, the FastText language model identifies the dominant language of each message. Non-English messages receive a ‘Language’ label and do not pass through the rest of the pipeline since the catfuzz and DistilBERT models can process English only. Each message is then labeled separately by the catfuzz model and the DistilBERT model, as described below. Finally, labels predicted by catfuzz and DistilBERT are used as input to a simple neural network that outputs a final set of label(s) for each message.

The catfuzz model

Catfuzz, named for “categorization with fuzzy string-matching” but also a homage to the second author’s cat, is a custom-built, rule-based, fuzzy string-matching model. It first tokenizes and lemmatizes patient messages with a customized version of spaCy’s en_core_web_md[3] pipeline and then converts them to lower-case. Stop words are not removed; however, a customized list of common words with similar spellings to search terms are removed to avoid fuzzy-matching them to search terms.

Each category of message labels includes a list of search terms that have been identified by clinical teams. Search terms may consist of one or multiple words. The catfuzz model searches for each category’s search terms in each message using RapidFuzz[4,5,6] for fuzzy string matching. If at least one of a category’s search terms matches to words in a message with an average match score based on Levenshtein distance of 87% or greater, then that message will receive that category label based on the catfuzz approach. Ranges of numbers can also be defined as search terms and are matched with regular expression patterns rather than fuzzy string matching.

Two types of secondary terms may then be defined for each search term: *context terms* and *exception terms*. If a context term *is not found* within *n* tokens from its search term and/or an

© 2024 Liu VX et al. *JAMA Network Open.*

exception term *is found* within *n* tokens, where *n* is refined per search term to achieve the best results, then the message is no longer considered a match. Negations ("no", "not") can also be looked for within 5 tokens before and after the search terms and applied as an option to the catfuzz approach. The catfuzz pipeline outputs binary category labels (0 if label applies or 1 if it does not) for each category for each message.

The DistilBERT model

Every message is also categorized with a DistilBERT[7] classification model. We domain-adapted the DistilBertTokenizer from Hugging Face's transformer package by fine-tuning it on approximately 1.5 million patient secure messages. With a fine-tuned tokenizer, we truncate and pads messages to 256 tokens. The 256 token limit is based on the maximum message length (subject plus body) of 1100 characters patients are allowed in the secure messaging platform; fewer than 0.8% of messages exceed 256 tokens.

For the DistilBERT classifier model, we built a Pytorch[9] neural network consisting of the DistilBertModel from Hugging Face's transformer package followed by a linear layer compressing BERT's 768 dimensions to 128, a ReLU[10] layer, a dropout layer that removes 30% of nodes for regularization, and a second linear layer that outputs the percent probability for each label. The model was trained on an NVIDIA A100 Tensor Core GPU with 40GB for 5 epochs, with a batch size of 16, a learning rate of 4×10^{-5}, a Binary Cross Entropy loss function, and the AdamW[11,12] optimizer. The training dataset (described below) of 20,893 patient-written messages was split into 60% train, 20% validation, and 20% test. The category label probabilities output by the trained DistilBERT classifier are binarized by mapping probabilities of 0.5 or greater to 1, and lower than 0.5, to 0 before being used as inputs into the neural network model.

© 2024 Liu VX et al. *JAMA Network Open.*

The neural network model

The catfuzz and DistilBERT predictions are input into a scikit-learn[8] MLPClassifier. The simple neural network's hidden layer size is equal to the number of categories and uses a ReLU activation function. For training, we used a learning rate of 0.001, the adam[12] optimization function, and L2 regularization with alpha=1×10^{-5}. Since we used 80% of our original 20,893 labeled messages to train and validate the DistilBERT classifier, we split the remaining 4,178 labeled messages into train (70%) and test (30%). Labels predicted by catfuzz and DistilBERT are used as input features, and the hand-labels assigned by nurses experienced with clinical topics in secure messages are used as targets when training. The trained neural network weighs the probability of a correct prediction by each of catfuzz and DistilBERT for a given category and outputs the final predicted category labels for the integrated pipeline.

Performance

The F1-scores for each secure message category range from 58% for Emergent (which was optimized based on clinician feedback to produce fewer false negatives and exhibits a recall of 81%) to 98% for Erectile Dysfunction and MPox. The macro F1-score across all categories for catfuzz alone is 87%, for DistilBERT alone is 88%, and for the final integrated pipeline (neural network) output is 88%. We chose to keep the catfuzz model in the integrated pipeline because it outperforms the DistilBERT pipeline in some categories (e.g., Controlled Substances, COVID19 Vaccine, Erectile Dysfunction, among others) and returns the message text that generates catfuzz's predicted label(s).

The dataset

The dataset used for training the DistilBERT and neural network models and validation of all models in the integrated pipeline comprised of 20,893 patient secure messages as of August 31, 2023. Messages were hand-labeled by two registered nurses based on guidelines defined

© 2024 Liu VX et al. *JAMA Network Open.*

by physicians and members of the regional Desktop Medicine team. The category support ranges from 486 messages (FIT Kit category) to 7040 (Medications category), with a median number of 962 labeled messages per category. It was not possible to create a dataset with an equal number of messages per category since patients commonly write about multiple topics, and increasing or decreasing the number of messages in one category will affect the counts of other categories.

eReferences

1. Joulin, Grave E, Bojanowski P, et al. Bag of Tricks for Efficient Text Classification. *arXiv:* 1607.01759. 2016.
2. Joulin, Grave E, Bojanowski P, et al. FastText.zip: Compressing text classification models. *arXiv:* 1612.03651. 2016.
3. Weischedel R, Palmer M, Marcus M, et al. OntoNotes Release 5.0 LDC2013T19. Web Download. Philadelphia: Linguistic Data Consortium, 2013.
4. Hyyrö H. A bit-vector algorithm for computing levenshtein and damerau edit distances. *Nordic J. of Computing.* Mar 2003;10(1):29–39.
5. Hyyrö H. Bit-parallel lcs-length computation revisited. *Proc. 15th Australasian Workshop on Combinatorial Algorithms (AWOCA 2004)*. 2004;08.
6. Wagner RA, Fischer MJ. The string-to-string correction problem. *J. ACM*. doi:10.1145/321796.321811. Jan 1974; 21(1):168–173.
7. Sanh V, Debut L, Chaumond J, et al. DistilBERT, a distilled version of BERT: smaller, faster, cheaper and lighter. *arXiv:* 1910.01108. 2020.
8. Pedregosa F, Varoquaux G, Gramfort A, et al. Scikit-learn: Machine Learning in Python. *JMLR*. 2011;12:2825-2830.
9. Paszke A, Gross S, Massa F, et al. *PyTorch: An Imperative Style, High-Performance Deep Learning Library*. Advances in Neural Information Processing Systems 32. 2019;8024-8035.
10. Agarap AF. Deep learning using rectified linear unites (relu). *arXiv:* 1803.08375. 2018.
11. Loshchilov I, Hutter F. Decoupled Weight Decay Regularization. *arXiv:* 1711.05101. 2019.
12. Kingma D, Ba J. Adam: A Method for Stochastic Optimization. arXiv: 1412.6980. 2017.

© 2024 Liu VX et al. *JAMA Network Open.*
